# Progranulin promotes hippocampal neurogenesis and alleviates anxiety‐like behavior and cognitive impairment in adult mice subjected to cerebral ischemia

**DOI:** 10.1111/cns.13810

**Published:** 2022-02-10

**Authors:** Siqi Sun, Jinlong Zhou, Zhongqi Li, Yuzi Wu, Hao Wang, Qi Zheng, Frank Adu‐Nti, Juan Fan, Yingfang Tian

**Affiliations:** ^1^ Key Laboratory of Ministry of Education for Medicinal Plant Resource and Natural Pharmaceutical Chemistry National Engineering Laboratory for Resource Developing of Endangered Chinese Crude Drugs in Northwest of China College of Life Sciences Shaanxi Normal University Xi’an China; ^2^ College of Life Sciences Shaanxi Normal University Xi’an China; ^3^ Key Laboratory of Modern Teaching Technology Ministry of Education Shaanxi Normal University Xi’an China

**Keywords:** anxiety, cerebral ischemia, hippocampus, learning and memory, neurogenesis, progranulin

## Abstract

**Aims:**

Cerebral ischemia can lead to anxiety and cognitive impairment due to the loss of hippocampal neurons. Facilitation of endogenous neurogenesis in the hippocampus is a potential therapeutic strategy for alleviating ischemia‐induced anxiety and cognitive impairment. Progranulin (PGRN), a secretory glycoprotein, has been reported to have a mitogentic effect on many cell types. However, it is not clear whether PGRN enhances hippocampal neurogenesis and promotes functional recovery.

**Methods:**

Adult male C57BL/6 mice were subjected to permanent middle cerebral artery occlusion (pMCAO) and injected intracerebroventricularly with recombinant mouse PGRN 30 min after pMCAO. Anxiety‐like behavior was detected by the open field and the elevated plus maze tests, and spatial learning and memory abilities were evaluated by Morris water maze. Neurogenesis was examined by double labeling of BrdU and neural stem cells or neurons markers. For mechanism studies, the level of ERK1/2 and AKT phosphorylation were assessed by western blotting.

**Results:**

Progranulin significantly alleviated anxiety‐like behavior and spatial learning and memory impairment induced by cerebral ischemia in mice. Consistent with the functional recovery, PGRN promoted neural stem cells (NSCs) proliferation and neuronal differentiation in the dentate gyrus (DG) after cerebral ischemia. PGRN upregulated the expression of phosphorylated ERK1/2 and Akt in the DG after cerebral ischemia.

**Conclusions:**

Progranulin alleviates ischemia‐induced anxiety‐like behavior and spatial learning and memory impairment in mice, probably via stimulation of hippocampal neurogenesis mediated by activation of MAPK/ERK and PI3K/Akt pathways. PGRN might be a promising candidate for coping with ischemic stroke‐induced mood and cognitive impairment in clinic.

## Graphical abstract

Progranulin alleviates anxiety‐like behavior and spatial learning and memory impairment induced by cerebral ischemia, which is due to stimulating hippocampal neurogenesis through activation of MAPK/ERK and PI3K/Akt signaling pathways.

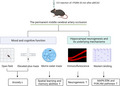

## 1 INTRODUCTION

Ischemia stroke is a cerebrovascular disease which is characterized by high fatality rate and high disability rate.^1^ In addition to physical impairments, stroke patients often endure post‐stroke anxiety and cognitive impairment including learning and memory deficit, resulting in significant decline in life quality.^2^ Previous studies in rodents reported that both middle cerebral artery occlusion (MCAO) and the bilateral common carotid artery occlusion (BCCAO) induced anxiety‐like behavior and spatial learning and memory impairment.^3^, ^4^ The hippocampus has considerable importance for mood and cognition.^5^, ^6^ Mood disorders and cognitive impairment caused by cerebral ischemia are closely related to neuronal damage in the hippocampus.^7^ In adult mammals, neurogenesis occurs in the subventricular zone (SVZ) of the lateral ventricle and the dentate gyrus (DG) of the hippocampus. A continuous proliferation of neural stem cells (NSCs) was found in the subgranular zone (SGZ) between the granule cell layer and the hilus.^8^ The ischemic injury stimulates the neurogenesis of SGZ, and the newly proliferated NSCs migrate and differentiate into mature neurons.^9^ Newborn neurons integrate into existing neural circuits and play an important role in functional recovery following cerebral ischemia.^9^ Therefore, many therapeutic strategies have been developed to promote hippocampal neurogenesis, which is promising to improve mood disorders and cognitive impairment induced by cerebral ischemia.^3^, ^9^, ^10^, ^11^, ^12^, ^13^

Progranulin (PGRN) is a secretory glycoprotein and abundantly expressed in the brain.^14^ PGRN has a mitogentic effect on many cell types including NSCs.^15^, ^16^ In recent decades, a few studies have showed that PGRN plays a positive role in hippocampal neurogenesis under several experimental conditions.^16^, ^17^, ^18^ PGRN deficiency decreased the proliferation of cultured hippocampal NSCs from embryonic mouse brains, which was rescued by the addition of exogenous PGRN.^16^ PGRN deficiency also exacerbated the suppressive effect of lipopolysaccharide on the adult hippocampal neurogenesis.^17^ Applying anti‐PGRN antibody blocked estrogen‐induced proliferation of adult hippocampal NSCs in vitro.^18^ PGRN was involved in enhancing hippocampal neurogenesis induced by voluntary exercise.^19^ Our previous study has reported that PGRN promotes neurogenesis in the subventricular zone of adult mice subjected to cerebral ischemia.^20^ However, whether administration of PGRN could attenuate mood disorder and cognitive impairment by promoting hippocampal neurogenesis after cerebral ischemia has not been investigated.

In this study, adult male C57BL/6 mice were subjected to the permanent middle cerebral artery occlusion (pMCAO) to induce cerebral ischemia, and received an intracerebroventricular (i.c.v.) administration of recombinant mouse PGRN (r‐PGRN) at 30 min after pMCAO. First, the effects of PGRN on anxiety‐like behavior and spatial learning and memory abilities of ischemic mice were examined. Then, we observed the effects of PGRN on NSCs proliferation and neuronal differentiation in the hippocampus after cerebral ischemia. Additionally, the molecular mechanism of PGRN's effects on neurogenesis after cerebral ischemia was tentatively explored.

## 2 MATERIALS AND METHODS

### 2.1 Animals

Adult male C57BL/6 mice weighting 20–25 g obtained from the Animal Center of Xi'an Jiaotong University were used in the current study. All animal procedures were licensed by the Shaanxi Normal University Ethics Committee and performed in accordance with the guidelines approved by the Animal Care and Use Committee of Shaanxi Normal University. All mice were maintained under standard conditions of 12 h/12 h light/dark cycle at 20–24°C. Food and water were available ad libitum.

### 2.2 Experimental design

**FIGURE 1:**
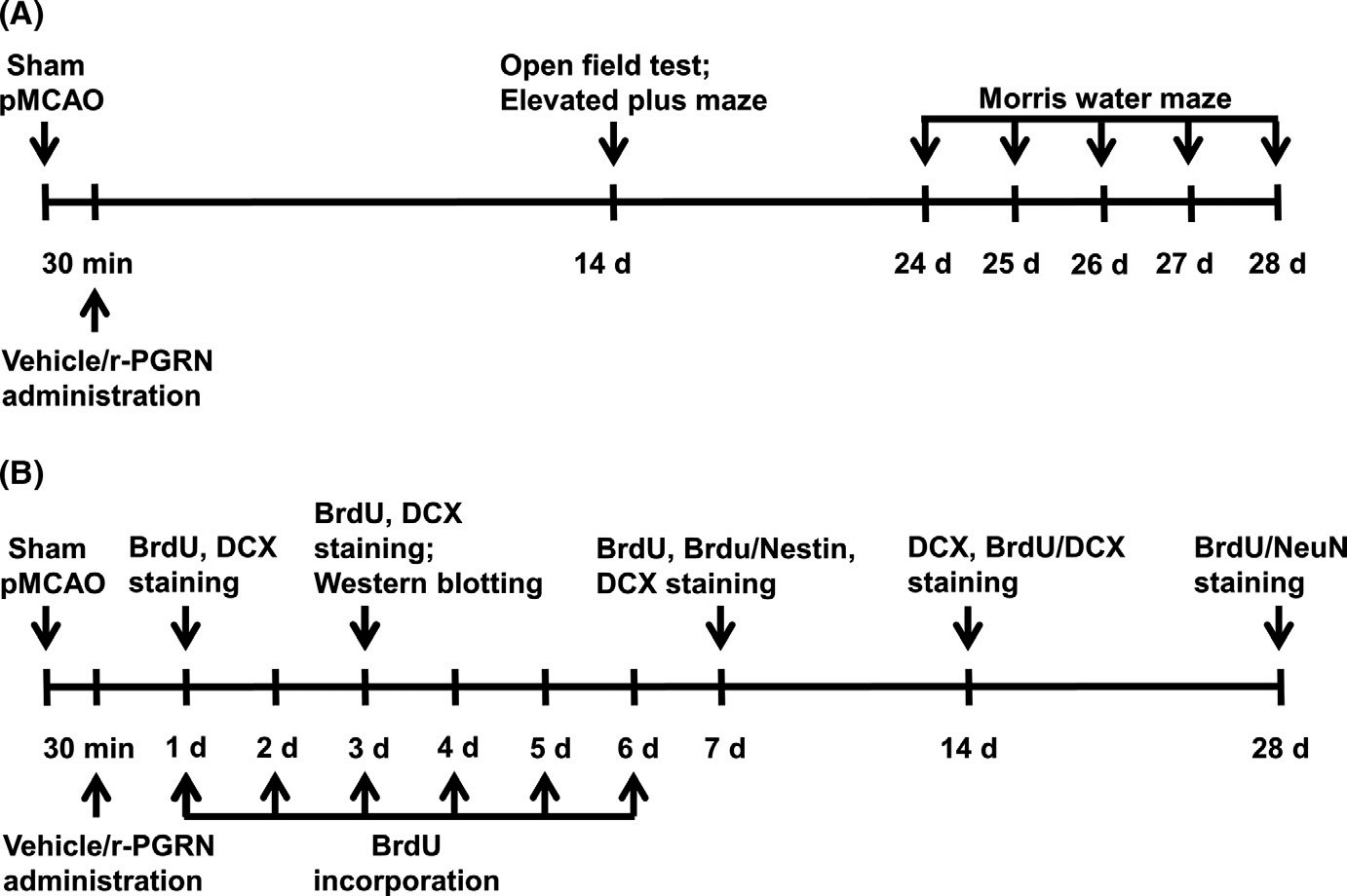
Schematic representation of experimental design. Cerebral ischemia was induced by pMCAO. Mice were randomly intracerebroventricular (i.c.v.) administered 1 ng recombinant mouse PGRN (r‐PGRN) or an equal volume of vehicle (0.01 M PBS, pH 7.4) 30 min after pMCAO. (A) Illustration of behavioral testing. Open‐field test and the elevated plus maze were performed 14 days after sham or pMCAO surgeries, and Morris water maze test was employed 24–28 days after surgeries. (B) Illustration of neurogenesis analysis. BrdU (50 mg/kg; i.p.) was administrated daily for 6 consecutive days (days 1 to 6) after sham or pMACO surgeries, and mice were then sacrificed for immunohistochemistry assays 1, 3, 7, 14, and 28 days after sham or pMCAO surgeries. Western blotting was conducted 3 days after sham or pMCAO surgeries. BrdU, 5‐bromo‐20‐deoxyuridine; DCX, doublecortin; pMCAO, permanent middle cerebral artery occlusion

A schematic of the experimental protocols is depicted in Figure 1. Briefly, mice were randomly assigned to the following three groups: the sham‐operated group, the pMCAO with vehicle‐treated group (pMCAO + Vehicle), and the pMCAO with PGRN‐treated group (pMCAO + PGRN). Intracerebroventricular injection of r‐PGRN (1 ng) or 0.01 M sterile phosphatase buffered saline (PBS) was performed 30 min after the pMCAO procedures. Mice in each group were then randomly divided into two subgroups for behavioral tests and neurogenesis detection, respectively.

Experiment 1 was designed to detect the effects of PGRN on mood and cognitive function following cerebral ischemia in mice (Figure 1A; *n* = 9 per group). Open‐field test and the elevated plus maze were performed to detect mice anxiety‐like behavior 14 days after sham or pMCAO surgeries. Morris water maze test was conducted to detect spatial learning and memory abilities 24–28 days after surgeries.

Experiment 2 was designed to detect the effect of PGRN on neurogenesis in SGZ and its underlying mechanisms following cerebral ischemia in mice (Figure 1B). According to previous reported methods,^21^ mice underwent daily 50 mg/kg intraperitoneal injections of 5‐bromo‐20‐deoxyuridine (BrdU; Sigma‐Aldrich) to label newborn cells, beginning 22 h following sham or pMCAO surgeries for 6 consecutive days. At 1, 3, 7, 14, and 28 days after sham or pMCAO surgeries, mice were sacrificed for immunohistochemistry assays (*n* = 5 per group). For the measurement of BrdU‐positive (BrdU^+^) cells, mice were sacrificed at 1, 3, and 7 days after sham or pMCAO surgeries. For the measurement of doublecortin (DCX)^+^ cells, mice were sacrificed at 1, 3, 7, and 14 days after surgeries. To detect the number of newborn NSCs (Nestin^+^/BrdU^+^ cells), immature neurons (DCX^+^/BrdU^+^ cells), and mature neurons (NeuN^+^/BrdU^+^ cells), mice were sacrificed at 7, 14, and 28 days after sham or pMCAO surgeries, respectively. Western blotting was conducted 3 days after sham or pMCAO surgeries (*n* = 3 per group).

### 2.3 Mice focal cerebral ischemia model

A mouse pMCAO model was performed as described previously.^22^ Briefly, mice were anesthetized intraperitoneally with sodium pentobarbital (50 mg/kg) and fixed on the operating table. A rectal probe was inserted to monitor body temperature, which was maintained at 37.0 ± 0.5°C with a heating pad. A midline skin incision was made in order to expose the right external carotid, internal carotid, and common carotid arteries. A 4–0 nylon monofilament with its tip rounded was carefully advanced into right internal carotid artery, until the origin of the middle cerebral artery was obstructed. Sham‐operated mice underwent the same operative procedure, but the middle cerebral artery was not occluded.

### 2.4 Recombinant mouse PGRN (r‐PGRN) administration

r‐PGRN (R&D Systems) was dissolved in 0.01 M sterile PBS and i.c.v. administrated using Hamilton syringe according to a previously described method.^23^ Briefly, 30 min after the pMCAO surgery, mice were placed on a stereotaxic apparatus (RWD Life Science) under anesthesia condition, and a microsyringe was used to give each mouse a single i.c.v. injection into the right lateral ventricle (0.5 mm posterior to bregma, 1.1 mm lateral to midline, and 2.5 mm vertically from the skull surface). r‐PGRN (1 ng in 2 µl PBS) or PBS (2 µl) was injected at a rate of 1 µl/min.

### 2.5 Behavioral testing

Mice were handled daily for 3 days before the behavioral test. At 14 days after sham or pMCAO surgeries, mice were tested first on the open‐field test followed by the elevated plus maze 6 h later. Before behavioral tests, all mice were allowed to habituate the testing room for at least 30 min. In all behavioral tests, animals' movements were recorded and analyzed using a CCD camera‐assisted motion tracking apparatus and software (VideoMot2, TSE Inst).

#### 2.5.1 Open‐field test

The apparatus was made of a polyvinyl chloride box (48 × 48 × 40 cm^3^) comprising of a white floor divided into 16 parts, and the most central 4 parts were set as the central area. The mice were individually placed in the central area of the floor, and allowed to explore freely in the box for 5 min. The total distance traveled in total area (indicator of locomotor activity) and the time spent in the central area (indicator of the anxiety level) were detected and analyzed.

#### 2.5.2 Elevated plus maze

The apparatus consisted of two open arms (28 × 6 cm^2^), two enclosed arms (28 × 6 × 15 cm^3^), and a central platform (6 × 6 cm^2^). The entire maze was raised 50 cm above the floor. Mice were placed individually into the center of the platform facing an open arm and allowed to explore freely the maze for 5 min. To assess the degree of anxiety, the percentage of time spent in the open arms ((time spent in the open arms / time spent in all arms) ×100%) and the number of entries into the open arms were detected and calculated.

#### 2.5.3 Morris water maze

A large circular pool (diameter 100 cm, depth 60 cm, and filled to a depth of 45 cm with water at 25 ± 1°C) was divided into four quadrants. A hidden circular platform (diameter 10 cm) at 1 cm below the water surface was placed in the center of one quadrant, providing the escape area. Mice were trained to discover the hidden platform for 4 consecutive days (spatial acquisition training days) with 4 training trials per day. During the training trials, mice were allowed to start from one random location in each of 4 quadrants and find the hidden platform within 60 s. If the mouse failed to find the hidden platform within 60 s, the experimenter guided them to reach it. Swimming speed, the escape latency, and the distance traveled to find the hidden platform were detected. On the fifth day, mice were subjected to a 60 s test (probe trial), in which the platform was removed. The time in the target quadrant (the location where the platform had been placed during the training tests) was recorded.

### 2.6 Immunohistochemistry and immunoflurescence

Mice were deeply anaesthetized and transcardially perfused with PBS, followed by 4% ice‐cold paraformaldehyde (PFA) at 1, 3, 7, 14, and 28 days after sham or pMCAO surgeries. Sixteen‐micrometer coronal brain sections were cut using a cryotome. To stain BrdU, the sections were treated with 2 mol/L HCl at 37°C for 30 min, and neutralized with 0.1 mol/L sodium borate (pH 8.5) for 10 min before blocking. After rinsing in PBS three times, sections were incubated in a blocking solution containing 0.5% Triton X‐100 and 5% bovine serum albumin for 1 h at room temperature. (1) For BrdU or DCX immunohistochemistry, the sections were incubated with primary antibodies (biotin‐labeled sheep anti‐BrdU antibody, 1:250; rabbit anti‐DCX antibody, 1:2000, Abcam) at 4℃ overnight. After washing, the sections were incubated with a streptavidin‐horseradish peroxidase complex or a biotin‐labeled goat anti‐rabbit IgG for 2 h at room temperature. The immunoreactivity was visualized with diaminobenzidine (DAB) and hydrogen peroxide. (2) For double immunoflurescence staining, the sections were incubated with rat anti‐BrdU antibody (1:250, Abcam), mouse anti‐nestin antibody (1:100, Millipore), rabbit anti‐DCX antibody (1:400, Abcam), and mouse anti‐NeuN antibody (1:200, Millipore). Appropriate secondary antibodies including Alexa fluor 488‐labeled donkey‐anti‐rat IgG (1:200, Jackson ImmunoResearch) and Delight 549‐labeled goat‐anti‐mouse or 549‐labeled goat‐anti‐rabbit IgG (1:200, Amyjet Scientific) were applied to visualize the immunofluorescence.

### 2.7 Cell counts and quantification

All images were acquired with a Zeiss microscope imaging system and a confocal laser scanning microscope (Olympus). Image J software (National Institutes of Health) was used to analyze the images. For quantification of BrdU^+^, BrdU^+^/nestin^+^, BrdU^+^/DCX^+^, and BrdU^+^/NeuN^+^ cells, cell counts were performed under 20× objective. Z‐stacked images were acquired every 1 μm throughout the section, and orthogonal reconstructions were performed along the xx, yy, and zz axes to confirm co‐localization. For analyses of DCX staining, images acquired under 40× objective were converted to 16‐bit black and white images and a fixed threshold was applied, then the percentage of area covered by the immunoreactive staining was calculated. The number of positive cells or the percentage of DCX^+^ area in the DG per animal were calculated and expressed as the mean value from eight sections of the entire hippocampus (approximately between −1.2 and −3.6 mm from the Bregma).

### 2.8 Western blotting

At 3 days after surgery, mouse hippocampal Cornu Ammonis 3 (CA3)/DG regions were collected, and lysed with ice‐cold homogenization buffer containing 2% protease inhibitor cocktail (Thermo Scientific). The samples containing equal amounts of protein were separated on 10% sodium dodecyl sulfate‐polyacrylamide gels (SDS‐PAGE), and transferred to PVDF membranes. After blocking with 5% nonfat milk and 0.1% Tween 20 in Tris‐buffered saline (TBS) at room temperature for 2 h, the membranes were incubated at 4℃ overnight with rabbit anti‐pERK1/2 (1:2500, Cell Signaling), rabbit anti‐ERK1/2 (1:2000, Cell Signaling), rabbit anti‐pAkt (1:2000, Cell Signaling), rabbit anti‐Akt (1:1000, Cell Signaling), and mouse anti‐β‐actin (1:10,000, Sigma). After washing, the membranes were incubated with peroxidase‐conjugated goat anti‐rabbit IgG (1:10,000, Sigma) or goat anti‐mouse IgG (1:10,000, Sigma) for 1 h at room temperature. The target protein signal was detected using a chemiluminescence detection kit (Pierce™ ECL western blotting Substrate, Thermo Scientific) and visualized on an electrophoresis image analyzer (Tianneng). β‐Actin was employed as the loading control. The band intensity was analyzed using Image J software. Quantification of densitometric values was calculated as follow: [(phospho‐protein/β‐actin)]/[total‐protein/β‐actin)] and expressed as ratio relative to the sham group.

### 2.9 Statistical analysis and quantification

All data are presented as mean ±standard error of mean (SEM). Statistical analyses were conducted using SPSS 10.0 (SPSS Inc.). The normality test was performed by the Shapiro‐Wilk test. The data in MWM training trial were analyzed using a two‐way repeated measure analysis of variance (ANOVA) with group and day. Statistical comparisons between two groups were performed using the independent two‐sample *t*‐test. Statistical comparisons among three groups were performed using one‐way ANOVA followed by the least significant difference (LSD) post hoc test. Differences were considered significant when *p* < 0.05.

## 3 RESULTS

### 3.1 Progranulin alleviates anxiety‐like behavior induced by cerebral ischemia in mice

**FIGURE 2:**
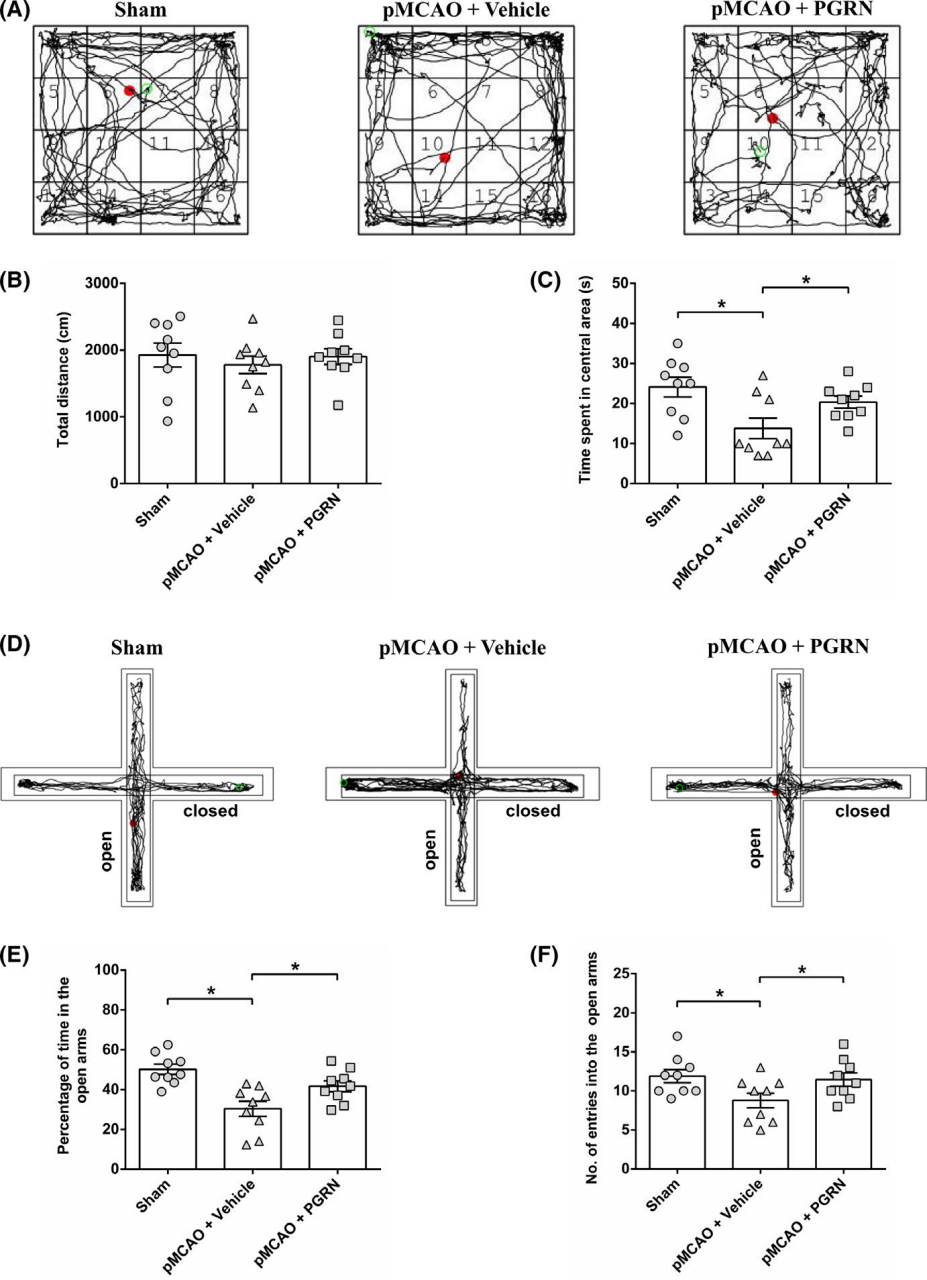
Intracerebroventricular delivery of 1 ng r‐PGRN alleviates cerebral ischemia‐induced anxiety‐like behavior. (A) Representative trajectory of mice in the open‐field test. (B) The total distance traveled in the open field for evaluating the locomotor activity. No difference in the locomotor activity was observed among the sham group, pMCAO + Vehicle group, and pMCAO + PGRN group. (C) The time spent in the central area for evaluating anxiety‐like behavior. (D) Representative moving traces of mice in the elevated plus maze. Anxiety‐like behavior was evaluated by measuring the percentage of time in the open arms (E) and the number of entries into the open arms (F) in the elevated plus maze. Data are presented as mean ± SEM, *n* = 9 per group. *p < 0.05

To determine whether administration of PGRN affects ischemia‐induced anxiety in mice, the anxiety‐like behavior was evaluated using the open‐field and the elevated plus maze tests (Figure 2A,D). The total distance traveled (indicator of locomotor activity) in the open filed had no difference among the three groups (all *p* > 0.05; Figure 2B). Meanwhile, mice in the ischemia (pMCAO + Vehicle) group presented a decrease in the time spent in the central area compared to the sham group 14 d after cerebral ischemia (*F*_(2, 24)_ = 5.516, *p* < 0.05; Figure 2C), indicating anxiety‐like effect of cerebral ischemia. PGRN administration attenuated ischemia‐induced anxiety‐like behavior, as shown by an increase in the time spent in the central area compared to the pMCAO + Vehicle group (*F*_(2, 24)_ = 5.516, *p* < 0.05; Figure 2C). In the elevated plus maze, mice in the pMCAO + Vehicle group exhibited a significant decrease in the percentage of time spent in the open arms (*F*_(2, 24)_ = 10.473, *p* < 0.05; Figure 2E) and the number of entries into the open arms (*F*_(2, 24)_ = 3.669, *p* < 0.05; Figure 2F) compared to the sham‐operated mice, suggesting the anxiety effects. The percentage of time spent in the open arms (*F*_(2, 24)_ = 10.473, *p* < 0.05; Figure 2E) and the number of entries into the open arms (*F*_(2, 24)_ = 3.669, *p* < 0.05; Figure 2F) increased significantly after the injection of PGRN compared to the ischemia mice. Taken together, the results indicate that PGRN treatment alleviated ischemia‐induced anxiety‐like behavior.

### 3.2 Progranulin alleviates spatial learning and memory impairment induced by cerebral ischemia in mice

**FIGURE 3:**
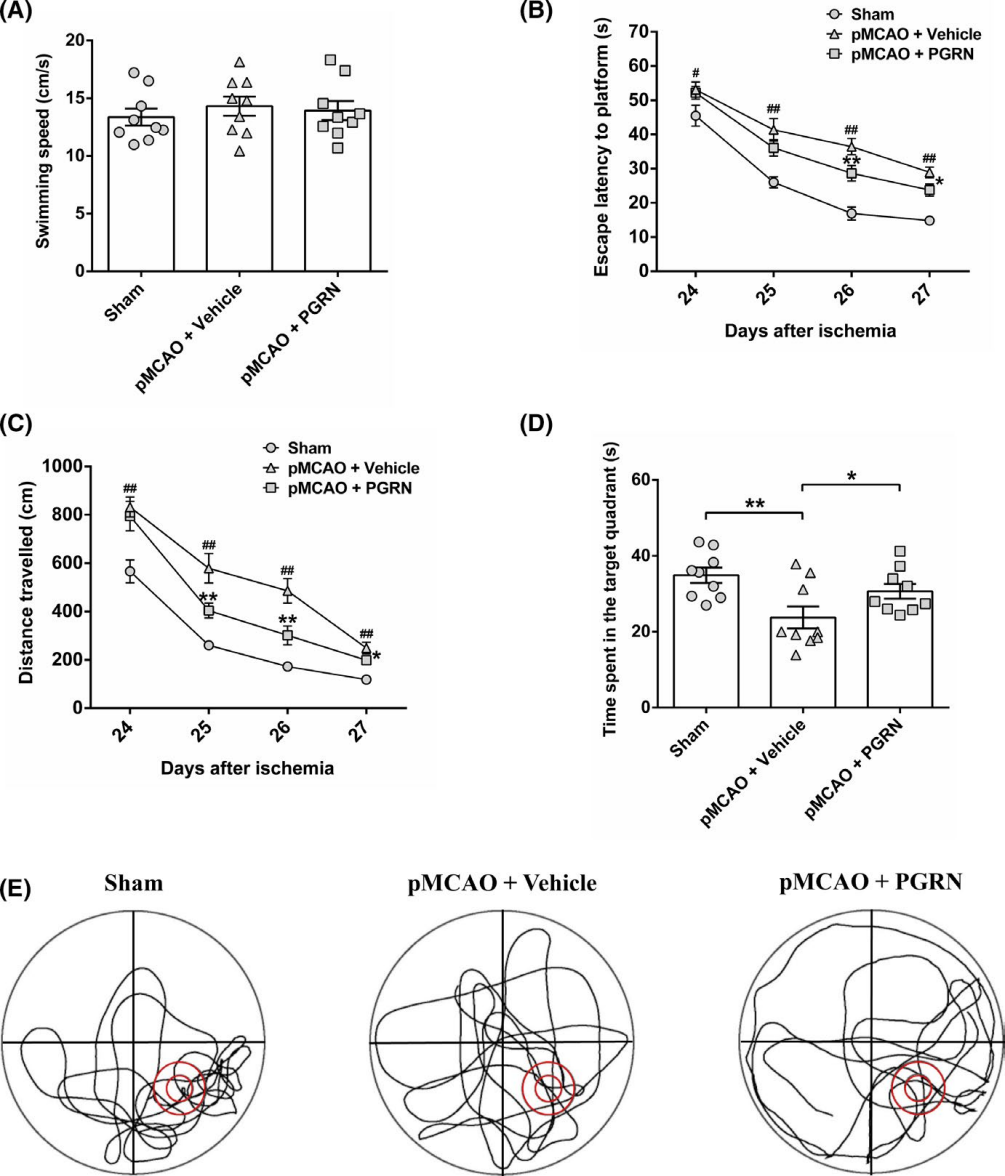
Progranulin (PGRN) administration alleviates cerebral ischemia‐induced spatial learning and memory impairment. (A) Swimming speed in the Morris water maze test for evaluating swimming abilities. No difference in swimming speed was observed among the sham group, pMCAO + Vehicle group, and pMCAO + PGRN group. Escape latency (B) and the distance traveled (C) to find the hidden platform measured during 24 to 27 days after cerebral ischemia in training trials. ^#^ *p* < 0.05, **^##^** *p* < 0.01 vs. sham group; ^*^ *p* < 0.05, ^**^ *p* < 0.01 vs. pMCAO + Vehicle group. (D) Time spent in the target quadrant 28 days after cerebral ischemia in probe trial. ^*^ *p* < 0.05, ^**^ *p* < 0.01. (E) Representative swimming traces of mice in probe trial. Data are presented as mean ± SEM, *n* = 9 per group

To investigate the effect of PGRN on cognitive deficits after cerebral ischemia, we conducted Morris water maze test to evaluate mouse spatial learning and memory abilities. Because swimming abilities impact the performance of Morris water maze test, swimming speed during the test was evaluated. The result showed there was no significant difference in swimming speed among the three groups, which indicated equivalent swimming abilities in Morris water maze test (all *p* > 0.05; Figure 3A). In the training session, group (*F*_group_ = 34.157, *p* < 0.001) and day (*F*_day_ = 98.992, *p* < 0.001) exerted significant effects on escape latency and the distance traveled to find the hidden platform during training days. The pMCAO + Vehicle group showed spatial learning and memory deficit compared to the sham group as indicated by the longer escape latencies (*F*_(2, 24)_ = 2.915, *p* < 0.05; *F*_(2, 24)_ = 11.495, *p* < 0.01; *F*_(2, 24)_ = 29.431, *p* < 0.01; *F*_(2, 24)_ = 21.245, *p* < 0.01 at training days 1, 2, 3, and 4, respectively; Figure 3B) and more distance traveled (*F*_(2, 24)_ = 8.091, *p* < 0.01; *F*_(2, 24)_ = 16.223, *p* < 0.01; *F*_(2, 24)_ = 17.856, *p* < 0.01; *F*_(2, 24)_ = 17.612, *p* < 0.01 at training days 1, 2, 3, and 4 respectively; Figure 3C) to find the hidden platform in training trials and shorter time in the target quadrant in probe trial (*F*_(2, 24)_ = 5.85, *p* < 0.01; Figure 3D,E). The pMCAO + PGRN group displayed a significant spatial learning and memory function restoration compared to the pMCAO + Vehicle group as shown by the shorter escape latencies from training days 3 to 4 (*F*_(2, 24)_ = 29.431, *p* < 0.01; *F*_(2, 24)_ = 21.245, *p* < 0.05 at training days 3 and 4, respectively; Figure 3B), reduced distance traveled from training days 2 to 4 (*F*_(2, 24)_ = 16.223, *p* < 0.01; *F*_(2, 24)_ = 17.856, *p* < 0.01; *F*_(2, 24)_ = 17.612, *p* < 0.05 at training days 2, 3, and 4 respectively; Figure 3C), and longer time spent in the target quadrant in probe trial (*F*_(2, 24)_ = 5.85, *p* < 0.05; Figure 3D,E). Overall, the results suggest that PGRN treatment alleviated the spatial learning and memory impairment induced by cerebral ischemia.

### 3.3 Progranulin promotes ischemia‐induced NSCs proliferation in the SGZ

**FIGURE 4:**
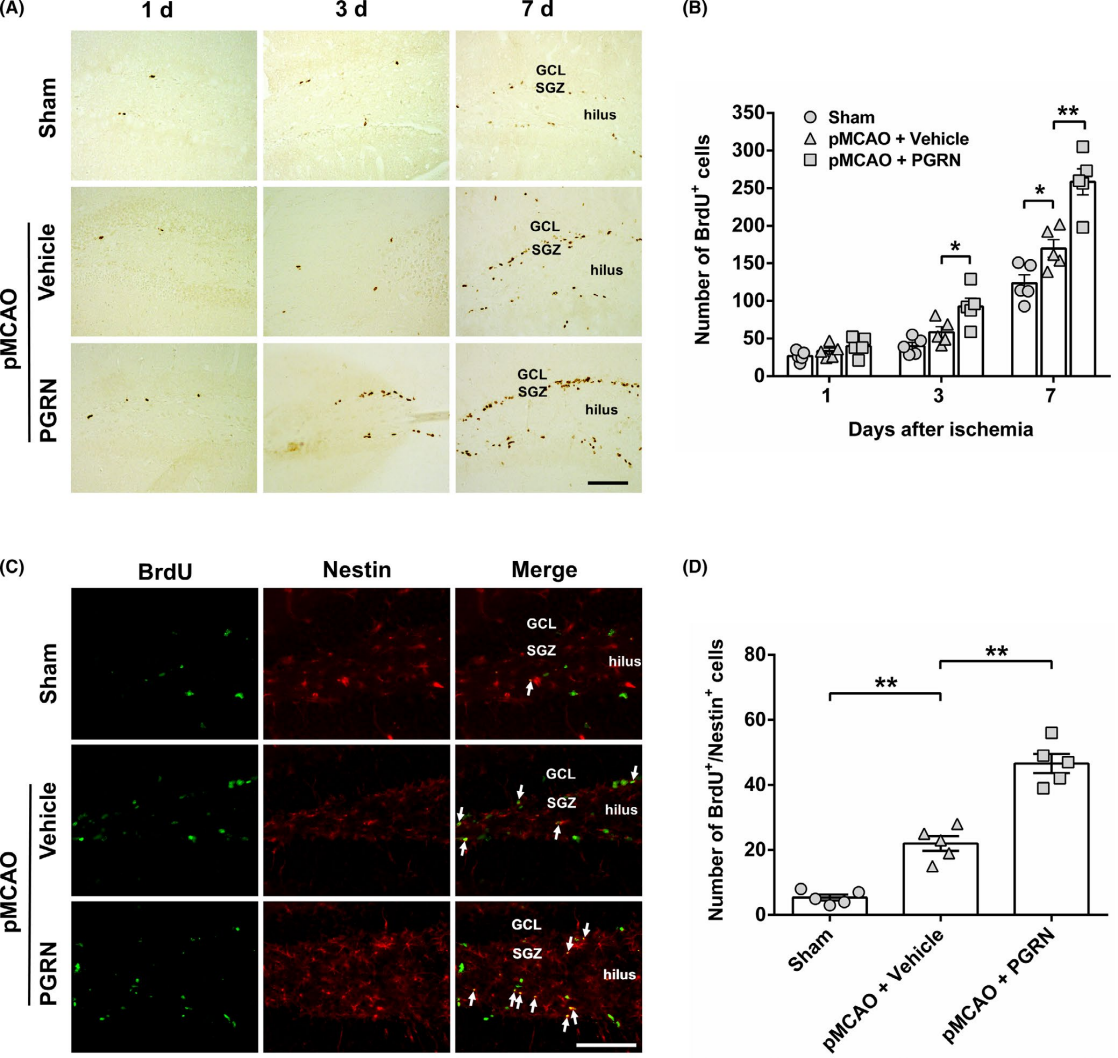
PGRN treatment promotes cerebral ischemia‐induced neural stem cells (NSCs) proliferation in the subgranular zone (SGZ) of hippocampal dentate gyrus (DG). (A) Representative photographs showed BrdU‐positive (BrdU^+^) cells in ischemic ipsilateral SGZ 1, 3, and 7 days after cerebral ischemia in the sham group, pMCAO + Vehicle group, and pMCAO + PGRN group. Positive cells are brown particles. Scale bar = 100 µm. (B) Quantitative analysis of the number of BrdU^+^ cells in the SGZ. (C) Representative photographs showed BrdU (green) and Nestin (red) double‐stained cells in ischemic ipsilateral SGZ 7 days after cerebral ischemia in the sham group, pMCAO + Vehicle group, and pMCAO + PGRN group. Arrows indicate BrdU^+^/Nestin^+^ cells. Scale bar = 50 µm. (D) Quantitative analysis of the number of BrdU^+^/Nestin^+^ cells in the SGZ. Data are presented as mean ± SEM, *n* = 5 per group. ^*^ *p* < 0.05, ^**^ *p* < 0.01. GCL, granular cell layer

To explore the effect of PGRN on NSCs proliferation in the SGZ following cerebral ischemia, we used BrdU to label newly proliferated cells. We first determined the time course of change in the number of BrdU^+^ cells in SGZ among the three groups. The pMCAO + Vehicle group exhibited an increasing tendency in the number of BrdU^+^ cells compared to the sham group 3 days after cerebral ischemia, but there was no statistical difference (*p* > 0.05; Figure 4A,B). A significant increase in the number of BrdU^+^ cells was detected in the pMCAO + Vehicle group compared to the sham group 7 days after cerebral ischemia (*F*_(2, 12)_ = 24.906, *p* < 0.05; Figure 4A,B). The results showed that ischemia‐induced cell proliferation in SGZ was time dependent. PGRN treatment further increased the number of BrdU^+^ cells compared to the pMCAO + Vehicle group at both 3 and 7 days after cerebral ischemia (*F*_(2, 12)_ = 10.639, *p* < 0.05; *F*_(2, 12)_ = 24.906, *p* < 0.01 at post‐ischemic day 3 and 7, respectively; Figure 4A,B). The results indicate that cerebral ischemia stimulates cell proliferation, and PGRN treatment further enhances ischemia‐induced cell proliferation in the SGZ.

Next, to assess whether these proliferating cells were indeed NSCs, double labelling of BrdU and nestin (a phenotypic marker of stem cell) was performed 7 d after cerebral ischemia. It turned out that cerebral ischemia significantly increased the number of BrdU^+^/nestin^+^ cells in SGZ compared to the sham group 7 days after cerebral ischemia (*F*_(2, 12)_ = 87.573, *p* < 0.01; Figure 4C,D). Compared with pMCAO + Vehicle group, the number of BrdU^+^/nestin^+^ cells in pMCAO + PGRN group was markedly increased 7 days after cerebral ischemia (*F*_(2, 12)_ = 87.573, *p* < 0.01; Figure 4C,D). Taken together, the results indicate that cerebral ischemia stimulates NSCs proliferation, and PGRN treatment further promotes ischemia‐induced NSCs proliferation in the SGZ.

### 3.4 Progranulin promotes ischemia‐induced NSCs neuronal differentiation in DG

To assess whether PGRN treatment regulates the NSCs differentiation in the hippocampus, double labeling of BrdU and cell markers (DCX, marker of immature neurons; NeuN, marker of mature neurons) was performed to label newborn immature and mature neurons.

**FIGURE 5:**
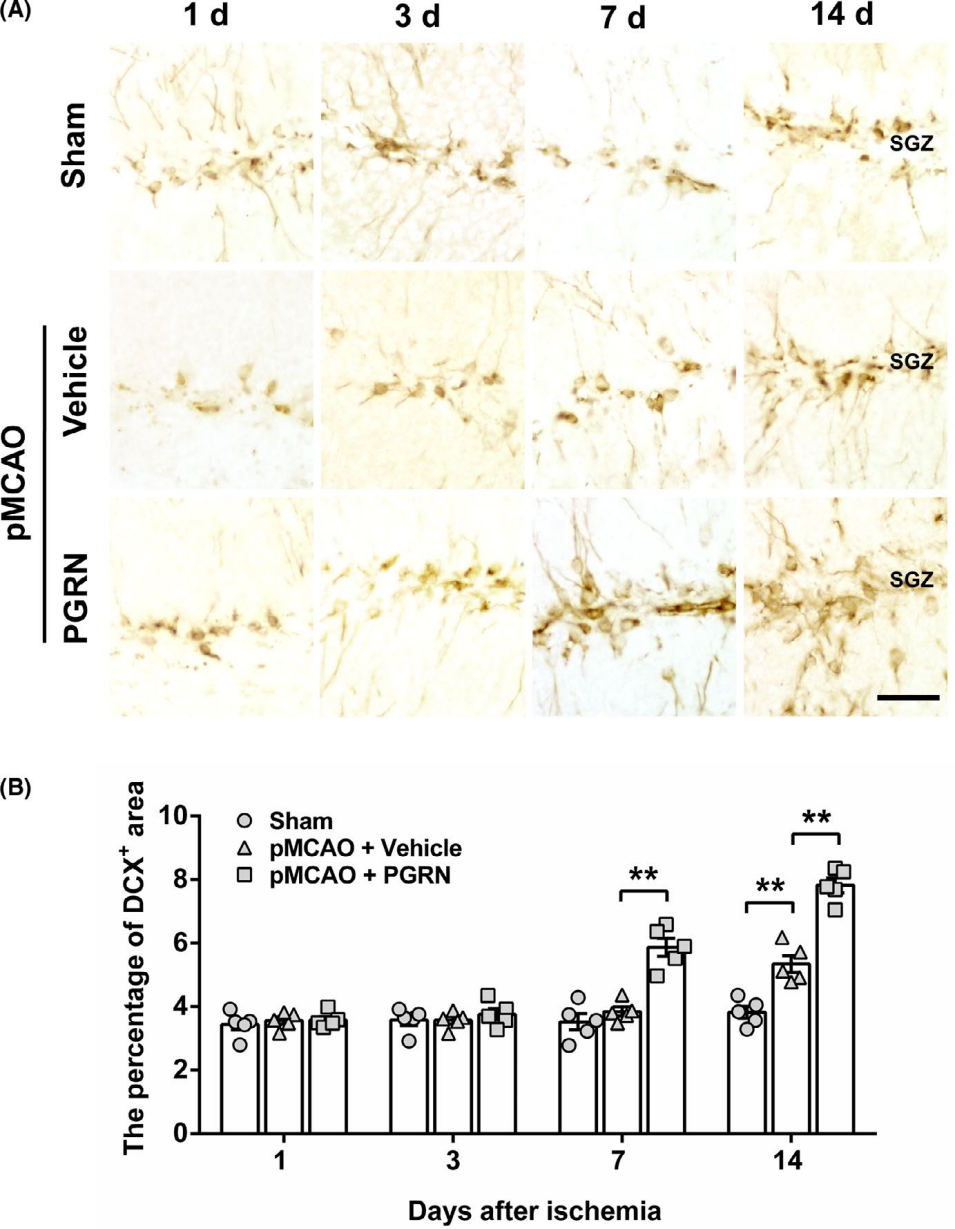
The effect of PGRN on immature neuron formation in the SGZ of mouse hippocampal DG after cerebral ischemia. (A) Representative photographs showed the DCX^+^ cells in ipsilateral SGZ 1, 3, 7, and 14 days after cerebral ischemia in the sham group, pMCAO + Vehicle group, and pMCAO + PGRN group. Positive cells are brown particles. Scale bar = 20 µm. (B) Time course of changes in the percentage of DCX^+^ area from the SGZ in different groups. Data are presented as mean ± SEM, *n* = 5 per group. ^**^ *p* < 0.01

We first detected the temporal patterns of DCX expression in SGZ among the three groups. Although no significant statistical difference was observed in DCX expression between the pMCAO + Vehicle group and the sham group 7 days after cerebral ischemia (*p* > 0.05; Figure 5A,B), the pMCAO + Vehicle group presented an increasing tendency compared to the sham group. DCX expression in the pMCAO + Vehicle group significantly increased compared to the sham group 14 d after cerebral ischemia (*F*_(2, 12)_ = 77.252, *p* < 0.01; Figure 5A,B). Compared to the pMCAO + Vehicle group, PGRN treatment significantly increased DCX expression in SGZ both 7 and 14 days after cerebral ischemia (*F*_(2, 12)_ = 28.347, *p* < 0.01; *F*_(2, 12)_ = 77.252, *p* < 0.01, at post‐ischemic days 7 and 14, respectively; Figure 5B). The results suggest that cerebral ischemia enlarges the population of immature neurons in the SGZ; PGRN treatment further increases the number of immature neurons following cerebral ischemia.

**FIGURE 6:**
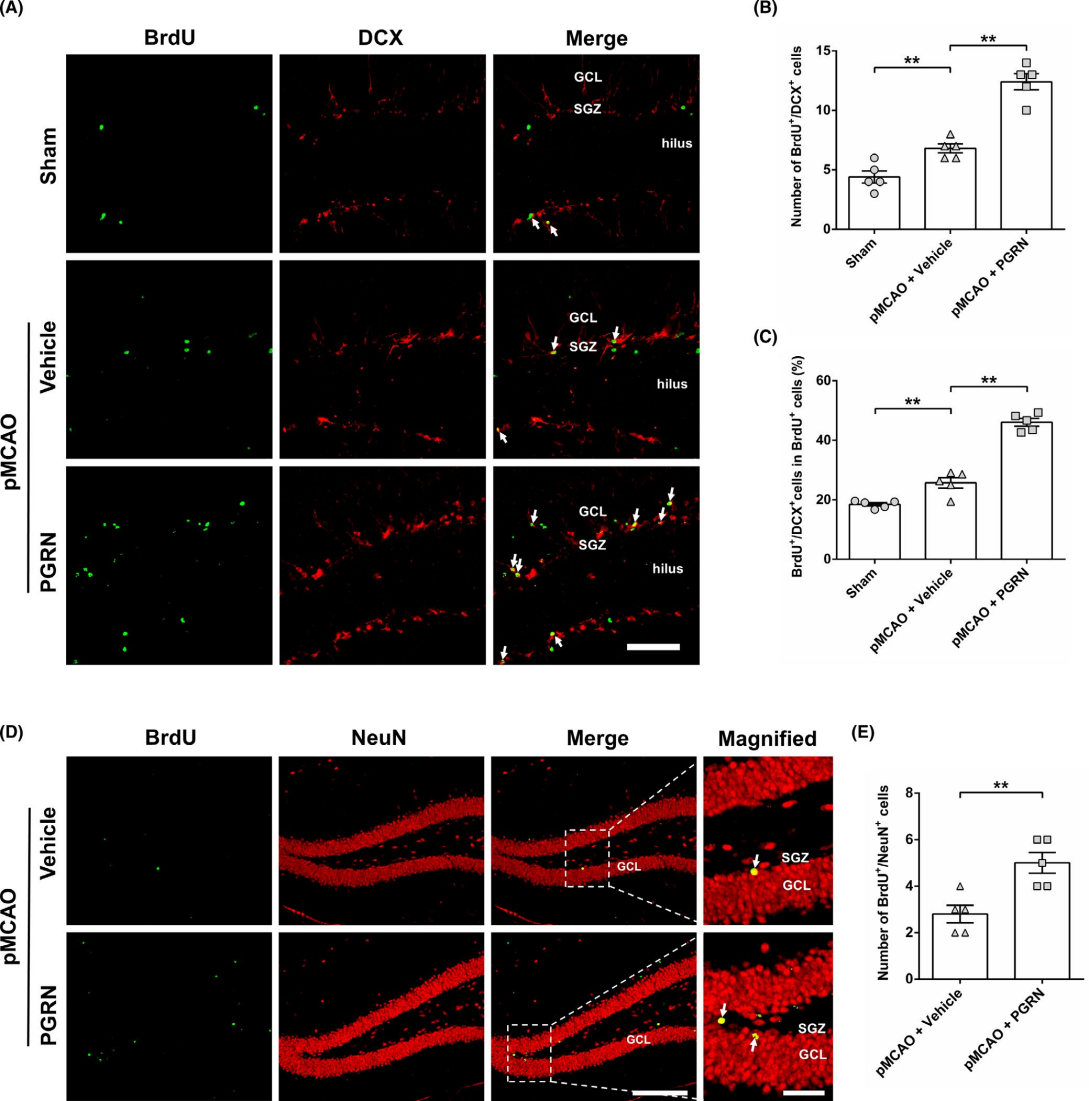
Progranulin (PGRN) promotes the differentiation of NSCs to immature and mature neuron in DG after cerebral ischemia. (A) Representative photographs showed BrdU^+^/DCX^+^ in SGZ 14 days after cerebral ischemia. BrdU^+^ cells are green particles, DCX^+^ cells are red. Arrows indicate BrdU^+^/DCX^+^ cells. Scale bar = 50 µm. Histograms showed the total number of BrdU^+^/DCX^+^ cells (B) and the percentage of BrdU^+^/DCX^+^ cells in the total BrdU^+^ cells (C) in the SGZ 14 days after cerebral ischemia. (D) Representative photographs showed BrdU^+^/NeuN^+^ cells 28 days after cerebral ischemia in DG. BrdU^+^ cells are green particles, NeuN^+^ cells are red. Arrows indicate BrdU^+^/NeuN^+^ cells. Scale bar in the low magnification = 200 µm, Scale bar in the high magnification = 50 µm. (E) Quantitative analysis of the number of BrdU^+^/NeuN^+^ cells in the DG in different groups. Data are presented as mean ± SEM, *n* = 5 per group. ^**^ *p* < 0.01

Next, we applied double labeling for BrdU and DCX to label the newly generated immature neurons in the SGZ 14 days after cerebral ischemia. A small number of BrdU^+^/DCX^+^ cells appeared in the sham group, while higher levels of these cells were observed in the pMCAO + Vehicle group (*F*_(2, 12)_ = 58.791, *p* < 0.01; Figure 6A,B). Compared to the pMCAO + Vehicle group, the number of BrdU^+^/DCX^+^ cells in the pMCAO + PGRN group was markedly increased 14 d after cerebral ischemia (*F*_(2, 12)_ = 58.791, *p* < 0.01; Figure 6A,B). The results demonstrate that cerebral ischemia stimulates the generation of immature neurons; PGRN treatment further promotes the generation of immature neurons following cerebral ischemia. Meanwhile, the percentage of BrdU^+^/DCX^+^ cells in BrdU^+^ cells was significantly increased in the pMCAO + Vehicle group compared to sham group 14 days after cerebral ischemia (*F*_(2, 12)_ = 122.69, *p* < 0.01; Figure 6C). PGRN treatment further increased the percentage of BrdU^+^/DCX^+^ cells in BrdU^+^ cells compared to the pMCAO + Vehicle group 14 days after cerebral ischemia (*F*_(2, 12)_ = 122.69, *p* < 0.01; Figure 6C), indicating cerebral ischemia stimulates NSCs differentiating into neurons, PGRN promotes ischemia‐induced neuronal differentiation of NSCs.

Newborn immature neurons further differentiate into mature neurons in DG granular cell layer. To estimate further the effect of PGRN on the differentiation of NSCs, the number of BrdU^+^/NeuN^+^ cells in DG was examined 28 days after cerebral ischemia (Figure 6). In DG, a few BrdU^+^/NeuN^+^ cells were observed in pMCAO+ Vehicle group 28 days after cerebral ischemia (Figure 6D). Compared to the pMCAO + Vehicle group, the number of BrdU^+^/NeuN^+^ cells in the pMCAO + PGRN group was significantly increased (*p* < 0.01; Figure 6E). In summary, these results suggest that PGRN promotes the differentiation of NSCs into neurons in DG after ischemia.

### 3.5 Progranulin treatment activates the MAPK/ERK and PI3K/Akt signaling pathways after cerebral ischemia

**FIGURE 7:**
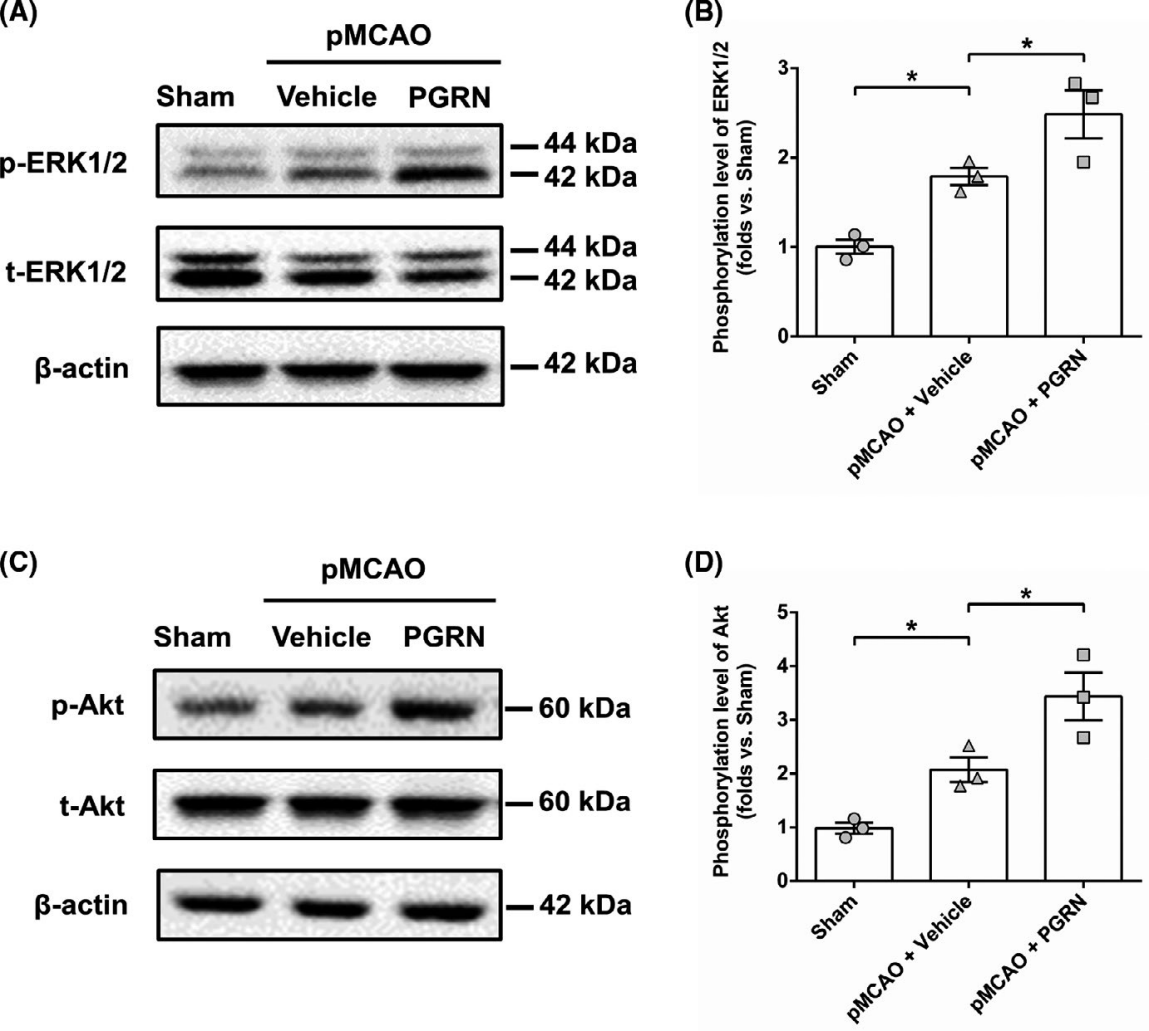
PGRN increased the level of ERK1/2 and Akt phosphorylation in DG 3 days after cerebral ischemia. Representative images of immunoblots using antibodies against p‐ERK1/2 (Thr‐202/Tyr‐204)/t‐ERK1/2 and p‐Akt (Thr‐308)/t‐Akt are shown (A and C), and quantitative analyses of ERK1/2 and Akt phosphorylation by densitometry are shown (B and D). Immunoblotting for actin reveals that equal amounts of proteins were loaded in each lane. Data are expressed as the ratio to the optical density value of sham‐operated animals. Data are presented as mean ± SEM, *n* = 3 per group. ^*^ *p* < 0.05

Activation of MAPK/ERK and PI3K/Akt signaling pathways in NSCs is crucial for the induction of adult neurogenesis.^24^ To explore whether PGRN's effect on ischemia‐induced neurogenesis in DG was through activation of MAPK/ERK and PI3K/Akt signaling pathways, we tested phosphorylated ERK1/2 and Akt in DG 3 days after cerebral ischemia by western blotting. The pMCAO + Vehicle group exhibited a significant increase in the expression of phosphorylated ERK1/2 (*F*_(2, 6)_ = 18.277, *p* < 0.05; Figure 7A,B and Figure S1) or Akt (*F*_(2, 6)_ = 17.352, *p* < 0.05; Figure 7C,D and Figure S1) compared to the sham group, and PGRN treatment further upregulated phosphorylation of ERK1/2 (*F*_(2, 6)_ = 18.277, *p* < 0.05; Figure 7A,B and Figure S1) or Akt (*F*_(2, 6)_ = 17.352, *p* < 0.05; Figure 7C,D and Figure S1) compared to the pMCAO + Vehicle group.

## 4 DISCUSSION

In this study, we provided the first evidence that PGRN treatment alleviated anxiety‐like behavior and spatial learning and memory impairment in adult mice subjected to pMCAO. Meanwhile, we demonstrated that PGRN treatment promoted the proliferation and neuronal differentiation of NSCs in hippocampus after cerebral ischemia. Additionally, PGRN treatment enhanced ischemia‐induced activation of MAPK/ERK and PI3K/Akt signaling pathways in DG. These results suggest that PGRN alleviates ischemia‐induced anxiety‐like behavior and reverses cognitive dysfunction in mice; hippocampal neurogenesis and activation of MAPK/ERK and PI3K/Akt signaling pathways may be involved in mediating the protective effects of PGRN.

In the present study, we used i.c.v. administration of 1 ng r‐PGRN after pMCAO to assess the effect of PGRN on ischemia‐induced behavioral deficits and hippocampal neurogenesis. Previous studies found that r‐PGRN at a dose of 1 ng via i.c.v. injection resulted in a reduction in the infarct volume and an improvement in neurological function in ischemic mice, and administration of r‐PGRN at lower doses (0.1 and 0.3 ng) or higher dose (5 ng) did not show the desired protective effect.^20^, ^23^ Our previous study also demonstrated that i.c.v. administration of 1 ng r‐PGRN promoted ischemia‐induced neurogenesis and neuronal differentiation in the SVZ.^20^ Based on these studies, we chose administration of r‐PGRN at a dose of 1 ng in this study, and found that 1 ng r‐PGRN significantly enhanced hippocampal neurogenesis and alleviated anxiety‐like behavior and spatial learning and memory impairment in ischemic mice.

The hippocampus is a key brain region for mood and cognition in mammals.^5^, ^6^ As a potential endogenous compensatory mechanism, cerebral ischemia has been shown to stimulate the proliferation of NSCs residing in SGZ, newly generated neurons form functional synapses and integrate into the existing hippocampal circuitry by 28 days, which contribute to improving functional recovery after cerebral ischemia.^9^, ^25^ As previous study reported, we also demonstrated that cerebral ischemia alone induced an increase in hippocampal NSCs proliferation (Figure 4) and neuronal differentiation (Figure 6). Several putative diffusible mitogens including growth factors, cytokines, and cell division modulators are known to be upregulated in the ischemic brain.^25^ These diffusible factors play a major role in promoting post‐ischemia neurogenesis.^25^ However, endogenous neurogenesis fails to produce adequate amounts of newborn neurons to facilitate the functional recovery.^9^ In this study, we found that PGRN administration has an improvement effect on ischemia‐induced anxiety‐like behavior 14 days after cerebral ischemia (Figure 2). By post‐ischemic day 14, newly generated neurons have not yet acquired the characteristics of mature neurons and integrated into hippocampal circuitry to play a functional role.^9^ Notable, we found that exogenous administration of PGRN enhanced ischemia‐induced neurogenesis in the SGZ, and increased the number of NSCs (Figure 4) and immature neurons in SGZ by post‐ischemic day 14 (Figure 5). NSCs has been reported to secrete growth factors (e.g., brain‐derived neurotrophic factor and vascular endothelial growth factor), which contribute to neuron repair and functional recovery by a broad range of neurotropic and neuroprotective effects.^12^, ^26^ Therefore, it could be concluded that PGRN facilitates cerebral ischemia‐induced NSCs proliferation in the SGZ, and NSCs could secrete growth factors which contribute to the reduction in anxiety‐like behavior after cerebral ischemia. In addition, we found that PGRN promoted NSCs differentiation into neurons and thereby resulted in more newborn mature neurons in the DG 28 days after cerebral ischemia (Figure 6). Combined with previous reports, our observations of improved spatial learning and memory after PGRN administration (Figure 3) may be attributable to more newborn neurons incorporated into the hippocampal network and formed functional synapses at post‐ischemic day 28. Our results support the hypothesis that enhanced neurogenesis contributes to functional recovery effect of PGRN following cerebral ischemia. However, it cannot be excluded that other potential effects of PGRN are also involved in the functional recovery. PGRN is a protein with multiple functions in the central nervous system including the regulation of neuroinflammation,^23^, ^27^, ^28^ neuronal survival,^27^, ^28^, ^29^ neurite growth, and synaptic plasticity.^30^, ^31^, ^32^, ^33^ A recent study has reported that PGRN not only attenuated sleep‐deprived–induced memory impairment and anxiety‐like behavior through enhancing neurogenesis in the SGZ but also through suppressing inflammation and restoring dendritic spine density in the hippocampus.^30^ Therefore, more studies are required to fully understand the mechanism behind it.

To study possible mechanisms through which PGRN induced increases in neurogenesis, selected proteins in the MAPK/ERK and PI3K/Akt signaling pathways were studied using western blotting. The MAPK/ERK and PI3K/Akt signaling pathways play a key role in adult hippocampal neurogenesis.^24^ The activation of MAPK/ERK and PI3K/Akt signaling pathways markedly enhances the cerebral ischemia‐induced hippocampal neurogenesis.^24^ PI3K inhibitor LY294002 and ERK kinase inhibitor U0126 inhibited hypoxia/reoxygenation‐induced increases in the proliferation in cultured mouse NSCs.^34^ Phosphorylation of ERK and Akt was significantly enhanced in the hippocampus and hemisphere on days 3 and 7 after the cerebral ischemia.^35^, ^36^ Consistent with these findings, we demonstrated that cerebral ischemia upregulated phosphorylation of ERK1/2 and Akt in the hippocampus (Figure 7). It has been reported that PGRN could activate these two pathways, resulting in increased expression of cyclin D1 and cyclin B and an enhanced proliferation rate in cancer cell.^37^, ^38^ Previous study showed that PGRN treatment increased the proliferation of cultured hippocampal NSCs, and PI3K inhibitor blocked this effect, suggesting that PGRN enhances NSCs proliferation and neurogenesis through activating the PI3K/Akt signaling pathway.^16^ Our previous study showed that PGRN may promote SVZ neurogenesis by activating the MAPK/ERK and PI3K/Akt signaling pathways.^20^ The current study revealed that administration of PGRN increased the level of phosphorylation of ERK1/2 and Akt induced by cerebral ischemia (Figure 7), suggesting that PGRN may promote ischemia‐induced hippocampal neurogenesis by activating the MAPK/ERK and PI3K/Akt signaling pathways.

Overall, this work is the first to demonstrate that PGRN enhances ischemia‐induced hippocampal neurogenesis through activation of MAPK/ERK and PI3K/Akt signaling pathways, thereby alleviating anxiety‐like behavior and the spatial learning and memory impairment following cerebral ischemia. Because of such biological functions, PGRN could be applied as a potential therapeutic agent to improve ischemic stroke‐induced mood disorder and cognitive impairment.

## CONFLICT OF INTEREST

The authors declare no conflict of interest.

## AUTHOR CONTRIBUTIONS

Siqi Sun, Juan Fan, and Yingfang Tian designed the research strategy, analyzed results, and wrote the manuscript. Siqi Sun, Jinlong Zhou, Zhongqi Li, Yuzi Wu, Hao Wang, Qi Zheng, Frank Adu‐Nti, Juan Fan, and Yingfang Tian contributed to conduct the experiments and to collect and analyze the data. All authors have read and approved the final manuscript.

## Supporting information

Figure S1

## Data Availability

The data that support the findings of this study are available from the corresponding author upon reasonable request.
