# Supplementary material for: Progranulin promotes hippocampal neurogenesis and alleviates anxiety‐like behavior and cognitive impairment in adult mice subjected to cerebral ischemia
Source: CNS Neurosci Ther. 2022 Feb 10;28(5):775–87. doi: 10.1111/cns.13810 (PMC8981488; doi:10.1111/cns.13810)
Supplement: Supplementary file 1 — Figure S1 [file CNS-28-775-s001.pdf]

Full unedited gel/blot for Figure 7A

p-ERK1/2

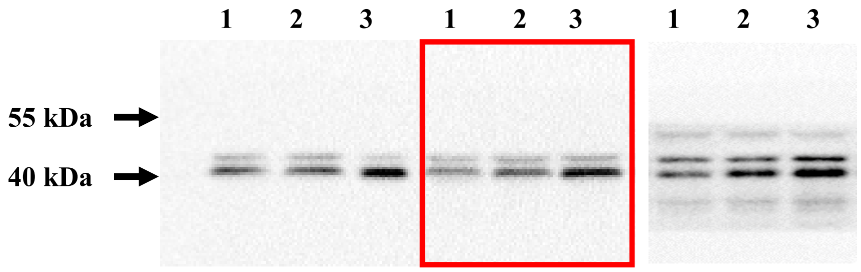

t-ERK1/2

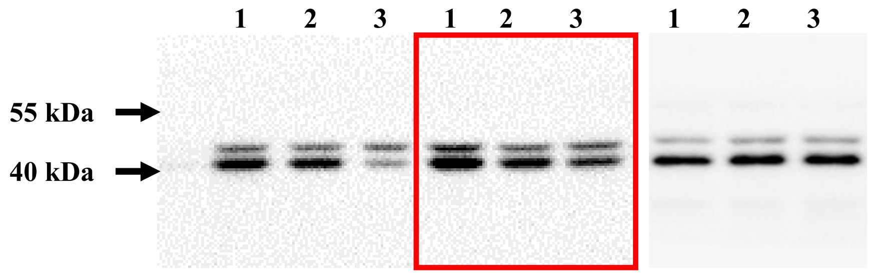

β-actin

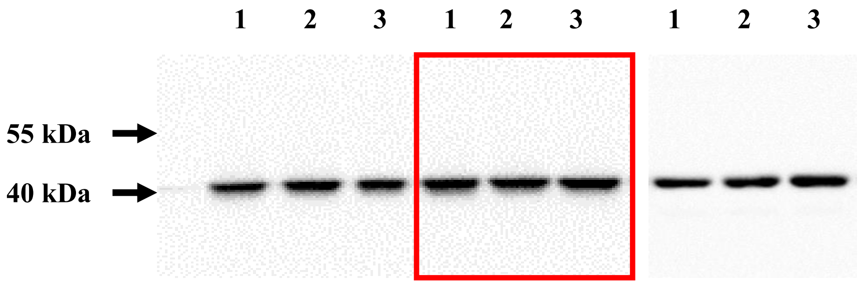

1 = Sham
2 = pMCAO + Vehicle
3 = pMCAO + PGRN

Full unedited gel/blot for Figure 7C

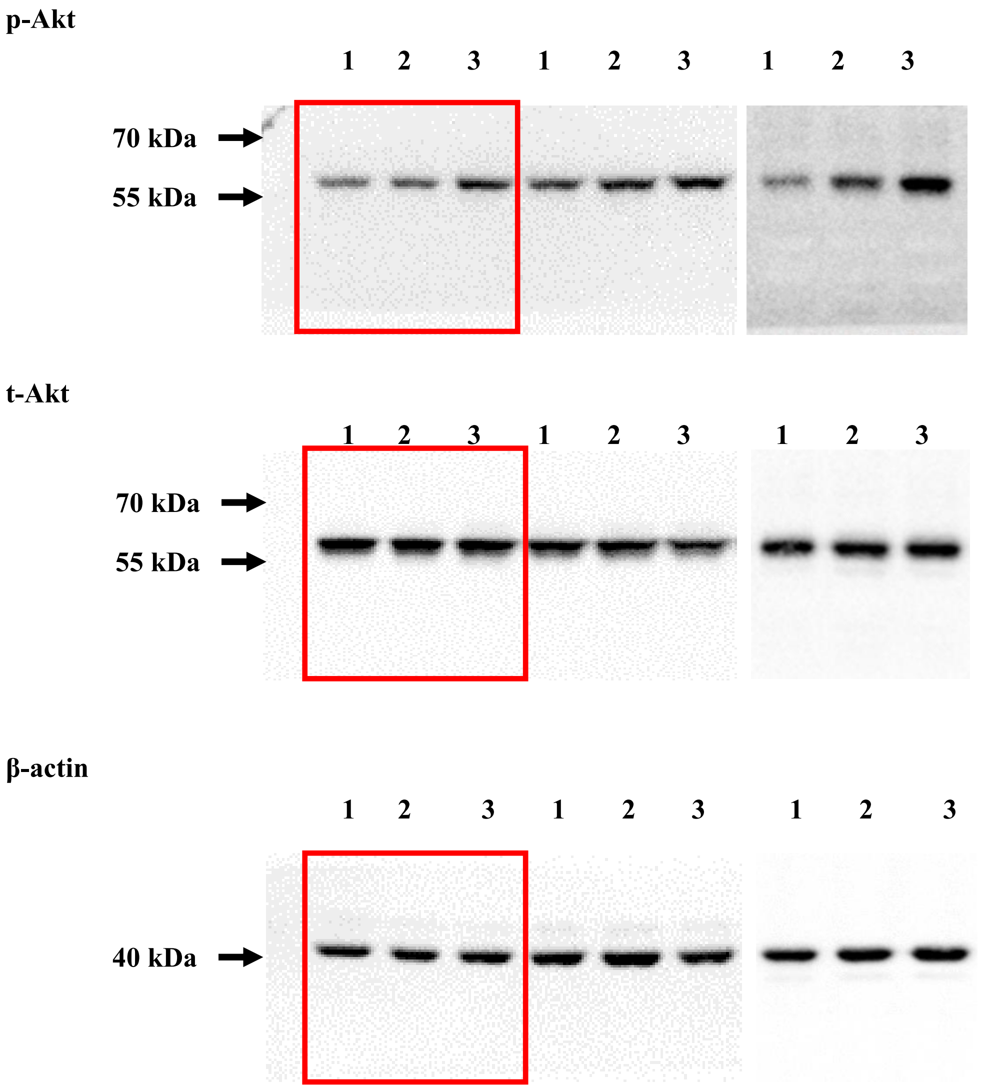

1 = Sham
2 = pMCAO + Vehicle
3 = pMCAO + PGRN
